# Photoresponsive Wettability in Monolayer Films from Sinapinic Acid

**DOI:** 10.1155/2013/915237

**Published:** 2013-11-05

**Authors:** Cleverson A. S. Moura, Douglas J. C. Gomes, Nara C. de Souza, Josmary R. Silva

**Affiliations:** Grupo de Materiais Nanoestruturados, Universidade Federal de Mato Grosso, 78600-000 Barra do Garças, MT, Brazil

## Abstract

Sinapinic acid is an interesting material because it is both antioxidant and antibacterial agent. In addition, when illuminated with ultraviolet light, it can exhibit the so-called photodimerization process. In this paper, we report on the investigation of monolayer films from 3,5-dimethoxy-4-hydroxycinnamic acid (sinapinic acid, SinA) deposited onto poly(allylamine hydrochloride), PAH, films. SinA monolayers were prepared by using the layer-by-layer (LbL) self-assembly technique. Adsorption kinetics curves were well fitted by a biexponential function suggesting that the adsorption process is determined by two mechanisms: nucleation and growth of aggregates. By using wetting contact angle analysis, we have found that SinA monolayers exhibit photoresponsive wettability under UV irradiation (365 nm); that is, wettability decreases with increasing UV irradiation time. The photoresponse of wettability was attributed to photodimerization process. This hypothesis was supported by the dependence of surface morphological structure and absorption on UV irradiation time. The mechanism found in the well-known transcinnamic acid crystals is used to explain the photodimerization process in SinA monolayers.

## 1. Introduction

Photoresponsive materials are an interesting class of new systems due to their potential application in devices such as microelectromechanical systems—MEMS [1]. In these systems, the control of properties such as wettability via one external stimulus is a key requisite for their application. In general, temperature [2], electric field [3], and light [4] have been used as stimulus for the wettability control in materials. One interesting material family, which responds to ultraviolet (UV) radiation, is the named cinnamic acid and its derivatives, which are widely used as model systems for photochemical reactions that can occur in condensate phase [5]. When the molecules of these materials—arranged in parallel stacking geometry—are exposed to ultraviolet light, they can undergo crystalline structure transformation as a result from photodimerization process [5]. This latter mechanism can cause morphological changes and therefore leading to a structural control of the films. A lot of experimental techniques have been employed to investigate films from cinnamic acid derivates under UV irradiation, for instance, UV absorption [6], infrared [7], Raman spectroscopy [8], X-ray structural analysis [9], and atomic force microscopy [10]. In particular, photodimerization has been studied on sinapinic acid by using subpicosecond time-resolved fluorescence spectroscopic [11]. Moreover, SinA has been investigated as a chromophore isolated model for photoactive yellow protein (PYP). In this study, the authors observed an unrelaxed ground-state intermediate in pump-probe signals by means of pump-dump probe spectroscopy [12]. All these studies are carried out in liquid or solid state phases; however there are not reports of studies of films prepared by layer-by-layer self-assembly technique. The use of the LbL technique can be interesting because it allows surface structure and thickness control, which leads to the buildup of the desired systems [13, 14].

In this paper, we report on the preparation and investigation of the photoresponse of wettability of SinA monolayers films irradiated by ultraviolet radiation (365 nm). For these studies, we have employed wetting contact angle measurements, atomic force microscopy, and UV-visible spectroscopy. SinA monolayers were deposited onto PAH layers, which were deposited previously on quartz substrate. PAH was used as a support because SinA molecules do not adsorb on bare quartz. 

## 2. Materials and Methods

**Figure 1:**
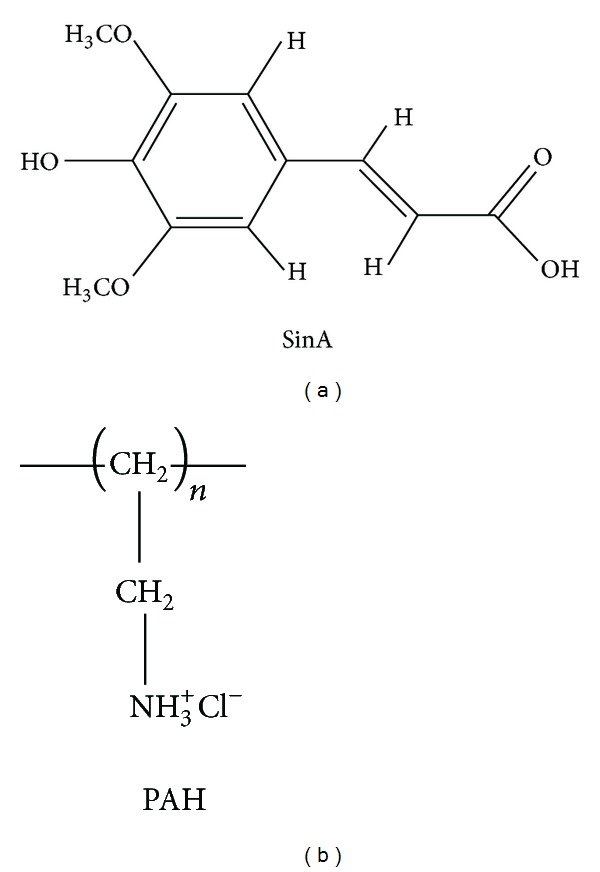
Chemical structures of sinapinic acid (SinA) and poly(allylamine hydrochloride) (PAH).

3,5-Dimethoxy-4-hydroxycinnamic acid (sinapinic acid, SinA) was purchased from Acros Organics (Figure 1). Poly(allylamine hydrochloride) (PAH) (MW ~15,000) was purchased from Sigma-Aldrich. All compounds were used as received.

For the adsorption kinetics experiments, the PAH monolayers were assembled by the immersion of the quartz substrate into PAH aqueous solution (0.5 mg/mL) for 3 min. Following, the system (PAH monolayer film + quartz substrate) was immersed into solution of SinA with methanol (10.0 mg/mL) for different immersion times (2–85 s) at room temperature (20°C). The immersion time is given by the addition of the times at each immersion step from the beginning of the experiment at 0 s. After each different immersion time, the SinA monolayers were dried under an air flow and their absorbance was measured by UV-visible spectroscopy spectrophotometer (Thermolab, Genesys 10). For the solutions of PAH, the pH was adjusted to 7.5 by adding NH_4_OH. For the experiment of irradiation of films with ultraviolet radiation, analyzes of AFM and for wetting contact angle, an immersion time of 45 s was used to prepare the samples. It should be noted that we call each of our films monolayer because they are formed by the same material in spite of using various steps of deposition with different immersion times to buildup them.

The exposures of the films to ultraviolet radiation were carried out by placing the samples into chamber with a Philips TL UV mercury lamp (6 W, 365 nm). The films were positioned 10 cm from the lamp. The surface morphology of the monolayers was studied with a NanoSurf Instruments atomic force microscope EasyScan *ΙΙ* in the tapping mode (256 × 256 pixels) under ambient conditions. A sample area of 10 **μ**m × 10 **μ**m was scanned and an image was acquired. The monolayer roughness and aggregate average height and diameter were determined using NanoSurf Instruments software. Wetting contact angles were measured with a homemade instrument in ambient conditions. Purified water droplets (volume of 5.0 **μ**L) were gently placed onto the film surfaces and the average values measured at six different locations of each sample were taken. In order to found the apparent surface energy, *γ*_*s*_^tot^, of the monolayers, we have used the following relation:

(1) γstot=γl(1+cos⁡θadv)22+cos⁡θrec+cos⁡θadv,

where *γ*_*l*_ is the surface tension of water, *θ*_adv_ is the contact angle of advance, and *θ*_rec_ is the receding contact angle [15]. Design and geometry optimization of SinA molecular were carried out in vacuum using MNDO method [16] implemented in ArgusLab 4.1 software [17].

## 3. Results and Discussion

### 3.1. Adsorption Kinetics

**Figure 2:**
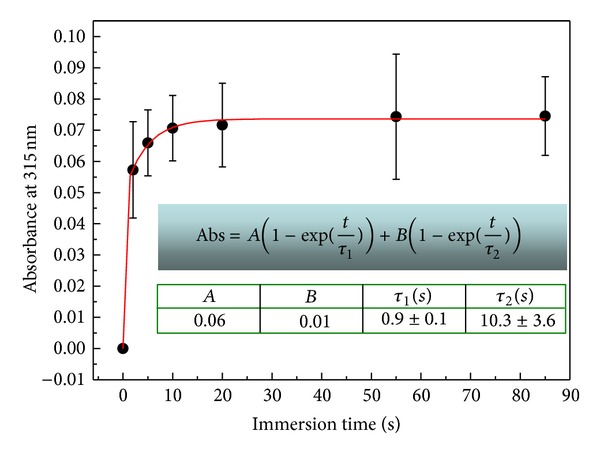
Growth kinetics followed by absorbance at 315 nm versus immersion time for a SinA monolayer.

Figure 2 depicts the adsorption kinetics for SinA monolayer films. UV-vis spectra of SinA present a well-defined absorption bands around 315 nm, which is attributed to *π* → π* transition in aromatic rings [18]. This value of absorption peak was used to the adsorption kinetics experiments.

The absorbance shows an increase as function of time and a plateau which is observed at constant time of ca. 10 s. This suggests that a whole monolayer was formed after 10 s. In this situation, the available sites for SinA molecules adsorption onto PAH films from solution are repelled by those already adsorbed with the same charge. 

The adsorption kinetics curve was fitted by an associated biexponential equation (inset Figure 2), where Abs is the absorbance, *A* and *B* are constants, *τ*_1_ and *τ*_2_ are the characteristic times, and *t* is the time [19]. 

Associated biexponential functions are commonly used for fitting kinetics processes such as photoinduced birefringence [20]. In general, this function represents two mechanisms in a kinetics phenomenon (fast and slow, resp.). For our results, the fast mechanism can be attributed to a fast adsorption mechanism in which the molecules near the PAH monolayer surface diffuse towards it filling the adsorption available sites. The slow mechanism arises from small amount of available sites after a few times and due to electrostatic repulsion between adsorbed molecules and those ones in solution [21].

### 3.2. Photoresponsive Wettability

**Figure 3:**
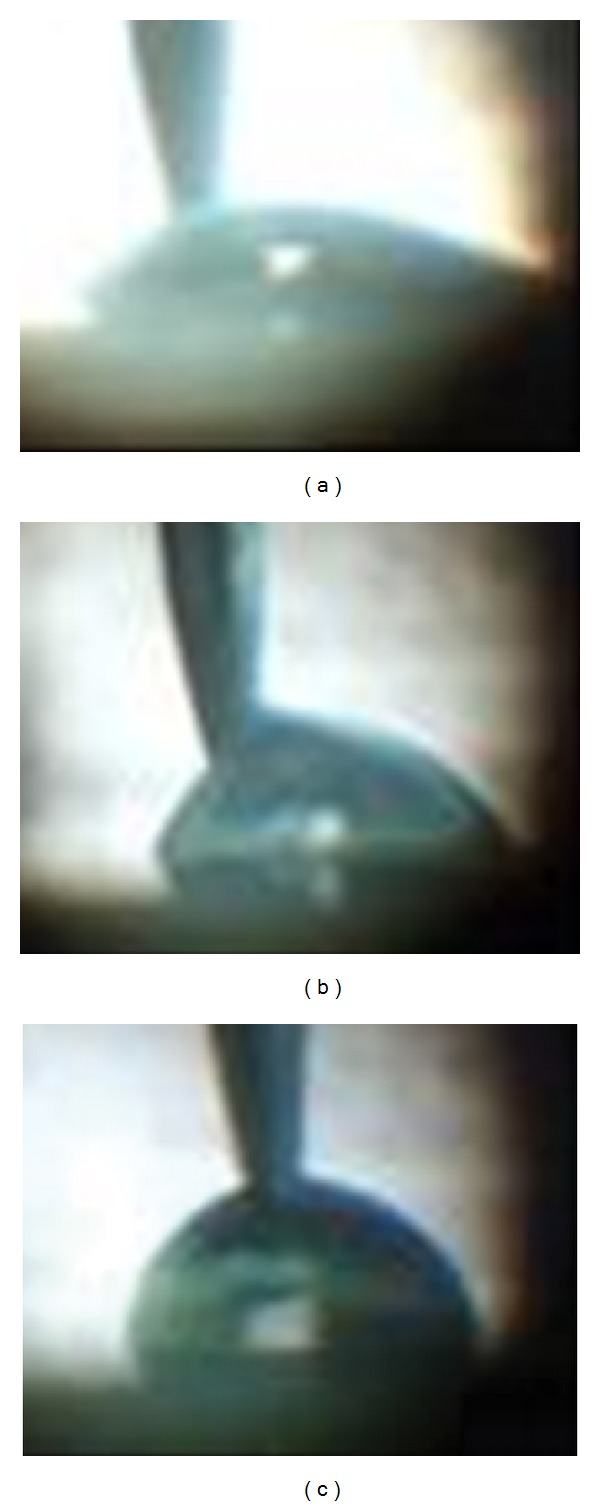
Water droplets on SinA monolayer during the advance wetting angle measurements: (a) as-prepared, (b) irradiated for 15 h, and (c) irradiated for 24 h.

In order to investigate the photoresponse of wettability from SinA monolayer under UV irradiation, we have performed wetting contact angle and obtained surface energies of SinA monolayers.  Figure 3 shows water droplets on a SinA monolayer, which were exposed to UV irradiation at 0 (as-prepared monolayer), 15, and 24 h.  Table 1 displays the values of wetting contact angles (advancing and receding) as well as the apparent surface energy values determined. 

It is observed from Figure 3 and Table 1 that the wetting contact angles increases with increasing UV irradiation time, whereas the apparent surface energy decreases. These finding indicates that the monolayer surface structure is converted from Wenzel one (Figure 3(a)) to a Cassie (Figure 3(c)) [22].

It is important to address that wetting contact angle and also apparent surface energy are determined by the chemical composition and surface morphological structure of material surface, which is associated to its roughness [19, 23]; the higher the roughness the lower the wettability, that is, low surface energy. On the other hand, photodimerization is associated to structural changes of material [11]. Then, we can hypothesize that the photoresponsive wettability exhibited by the SinA monolayer under UV irradiation, could occur due to a photodimerization process, which leads with structural alterations and, consequently, to a higher roughness of SinA monolayers. 

### 3.3. Surface Morphological Structure Analysis

**Figure 4:**
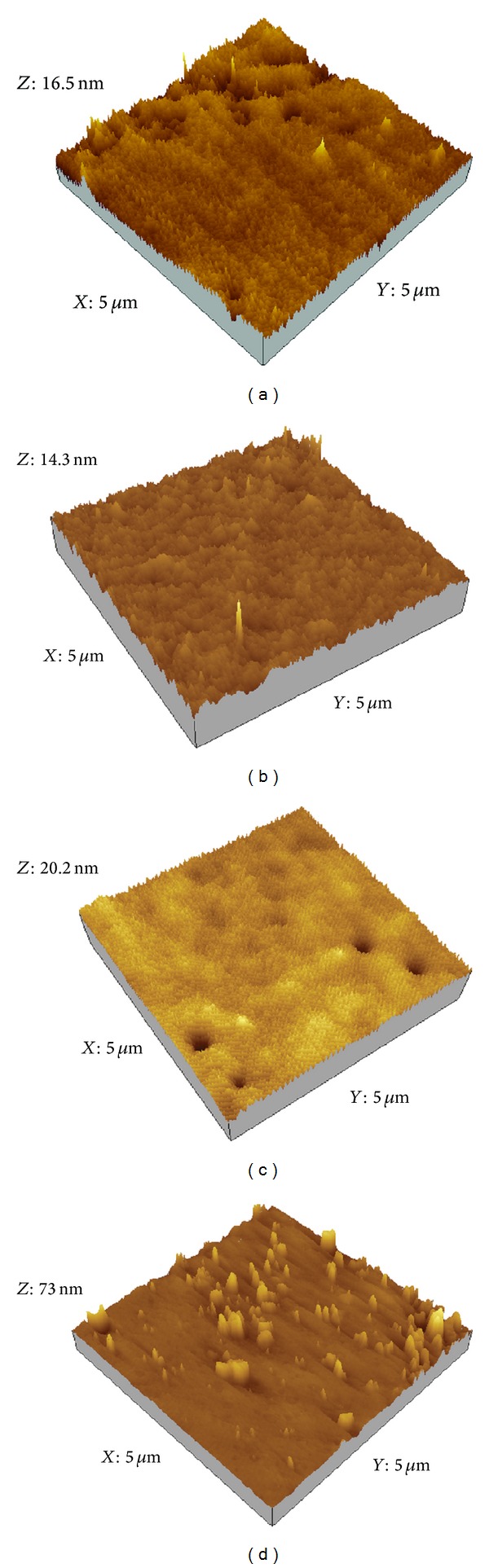
AFM images for (a) PAH monolayer onto quartz substrate, (b) SinA monolayer film onto PAH monolayer without UV irradiation (c) SinA monolayer after UV irradiation for 15 h, and (d) SinA monolayer after UV irradiation for 24 h.

In order to examine the hypothesis that increasing the roughness could decrease the wettability of SinA monolayers, we have carried out atomic force microscopy analysis.  Figure 4 shows the AFM image of a SinA monolayer film AFM images for (a) PAH monolayer onto quartz substrate, (b) SinA monolayer film onto PAH monolayer without UV irradiation (c) SinA monolayer after UV irradiation for 15 h, and (d) SinA monolayer after UV irradiation.

As shown in the Figure 4, we can observe that the surface morphological structure is formed by rod-shaped aggregates. This finding was expected since structural changes due to photodimerization have been observed for monolayers of 4-(amyloxy) cinnamic acid deposited on Au substrate [24]. AFM images reveal that the PAH film surface is very smooth, with roughness ca. 1 nm and aggregate free (Figure 4(b)). Then, the contribution of surface morphology structure of PAH film surfaces on SinA monolayer morphological structure could be ruled out. From Table 2, we observe that RMS roughness increase with increasing UV irradiation time. This result supports the hypothesis of increases in the roughness of SinA monolayer as the origin of the decreasing wettability of SinA monolayer (Table 1) and the transition from Wenzel surface to Cassie one. 

### 3.4. UV-Vis Analysis

**Figure 5:**
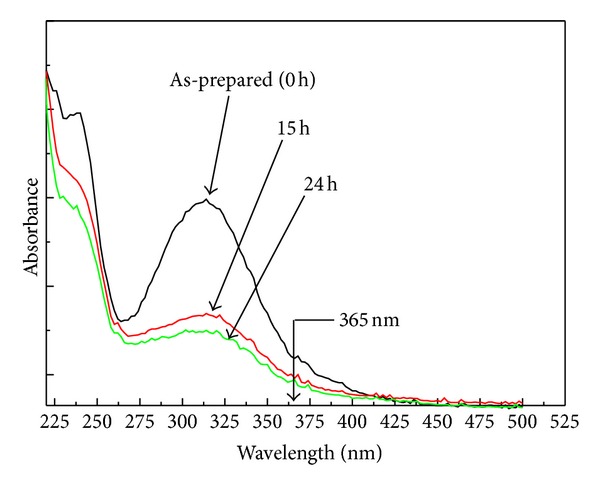
UV-visible spectra for as-prepared SinA monolayer and irradiated by UV radiation (365 nm) for 15 and 24 h.

In order to corroborate the photodimerization hypothesis of SinA under UV irradiation, we have carried out analysis of UV-vis spectroscopy.  Figure 5 shows the UV-visible spectra for SinA monolayer, which were submitted to different UV irradiation times. We can observe a decrease of absorbance as a function of the irradiation time. This behavior was also noted by Davaasambuu et al. [25] being attributed to a decrease in monomer concentration as a result from photodimerization process. 

Our result is consistent with those found from AFM (Section 3.3) and wettability contact angle analysis (Section 3.2). Therefore, it is reasonable suggesting that a photodimerization process is responsible by the photoresponse of wettability of the SinA monolayer under UV irradiation.

### 3.5. Photodimerization Mechanism

**Figure 6:**
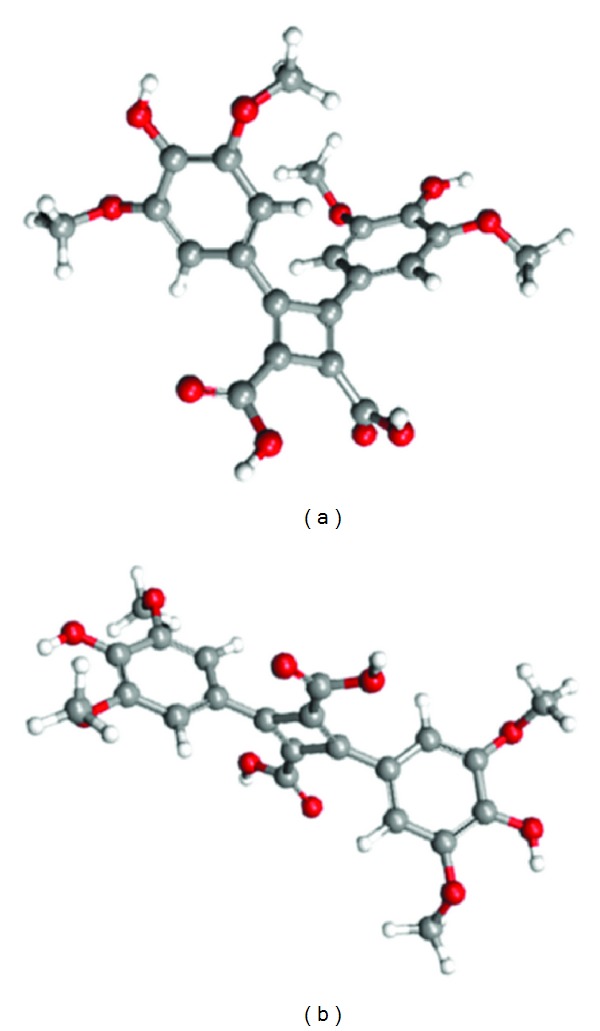
Possible molecular structures of dimmers formed from photodimerization of SinA from (a) head-tail arrange and (b) head-head arrange. Grey balls represent carbons, red balls represent oxygens, and white balls represent hydrogens. The structures were optimized by using the MNDO method.

In a study of self-assembled monolayers from 4-(amyloxy)cinnamic acid, Xu et al. [24] suggested that a photodimerization can arise from a molecular photoexcitation, which leads to a short-term lattice instability. This process would place one molecule close to a neighbor producing a more favorable molecular orientation and then leading to photodimerization reaction. Unfortunately, our results do not allow stating if this mechanism occurs in the SinA monolayer. On the other hand, similarly to photodimerization, which occur in transcinnamic acid [7], we can suggest two possible forms of dimer which can occur in the SinA monolayers after UV irradiation (a) head-head and (b) head-tail, as showed in Figure 6. 

Our results still do not allow stating which dimer form occur after UV irradiation of the SinA monolayer. A way of clarifying this point would be using the method proposed by Atkinson et al., which is based on vibrational spectroscopy [7].

## 4. Conclusion

We have prepared for the first time SinA monolayer films using PAH monolayer films as a support layer. Though adsorption kinetics experiments, the immersion time of saturation was found to be ~10 s. In addition, we have found that the growth process consists of two mechanisms, a fast one and another slow, which were associated with a fast adsorption limited by diffusion and an electrostatic repulsion, respectively. We have found that the wetting contact angle of SinA films increases with increasing UV irradiation time. This suggests that a photodimerization process plays an important role in the photoresponse of wettability. This hypothesis was corroborated by the surface morphological structure changes and decreasing in electronic absorption observed for the SinA monolayers as a function of irradiation time. In summary, monolayers films from cinnamic derivates—prepared by the LbL self-assembly technique—may be useful for future studies not only on photoresponse of wettability but also on elementary ablation, ionization processes. 

## Figures and Tables

**Table 1:** Values of wetting contact angles and apparent surface energy for as-prepared SinA monolayer and under different UV irradiation times.

Time (h)	*θ*_av_ (degree)	*θ*_rec_ (degree)	*γ*_*s*_ (mJ/m^2^)
0	42.2	34.6	35.6
15	72.3	37.8	30.7
24	100.1	43.7	23.6

**Table 2:** Mean diameter, root-mean-square (RMS) roughness for SinA monolayer at different times of UV irradiation.

Time (h)	RMS roughness (nm)
0	0.58
15	1.47
24	4.54
